# The mediating effect of math self-efficacy on the relationship between parenting style and math anxiety

**DOI:** 10.3389/fpsyg.2023.1197170

**Published:** 2023-06-09

**Authors:** Chao Wang, Xian Li, Hui-jiao Wang

**Affiliations:** ^1^Department of Psychology, Huzhou University, Huzhou, China; ^2^Department of Psychology, Neuroscience and Behaviour, McMaster University, Hamilton, ON, Canada

**Keywords:** math anxiety, parenting style, math self-efficacy, emotional warmth, rejection (psychology)

## Abstract

The present study aims to investigate the associations among math self-efficacy, parenting style, and math anxiety in primary school children. The sample comprised 400 participants, aged between 10 and 11 years old, from an elementary school in China. Participants completed three self-reported questionnaires on math anxiety, parenting styles and math self-efficacy. The results revealed that rejection was strongly and positively correlated with math anxiety, while emotional warmth was negatively related to math anxiety. Interestingly, math anxiety was found to be related to rejection, with math self-efficacy playing a mediating role in this relationship. Conversely, math self-efficacy played a mediating role in the relationship between parenting styles and math anxiety, while over protection exhibited no significant correlation with math anxiety. The study also showed that gender differences existed in the level of math anxiety and math self-efficacy, with boys exhibiting lower math anxiety and higher math self-efficacy than girls. These results provide important insights into the development and treatment of math anxiety in primary school children. Specifically, parents and educators should focus on enhancing children’s math self-efficacy beliefs, while adopting a parenting style characterized by emotional warmth and low levels of rejection.

## Introduction

Mathematics is important for the development of individuals and countries. Mathematics affected individuals’ decision making in daily activities (Ghazal et al., 2014), such as personal health (Huizinga et al., 2008), retirement savings (Banks and Oldfield, 2007), career choices (Levy et al., 2021). At the national level, improving science, technology, engineering, and mathematics (STEM) education is the key to economic growth and security (Xie et al., 2015; Bacovic et al., 2022). Additionally, the development of STEM fields depend on strong mathematical skills (Falloon et al., 2020; Yalçın, 2022). Hence, effectively improving mathematics performance had become an important concern for learners, educators and researchers (Ramirez et al., 2018).

### Math anxiety

Anxiety is one of most common negative emotions experienced by elementary school children (Kaskens et al., 2020; Raccanello et al., 2022). As an aspect of anxiety, math anxiety is a significant problem among primary school children (Dowker et al., 2016) and has attracted researchers’ attention on this topic. Researchers have observed that many people are afraid of math (Dreger and Aiken, 1957). Ashcraft (2002) defined math anxiety as the emotional responses of tension, apprehension, and fear that individuals experience when engage in math-related tasks, emphasizing the unpleasant experience of math anxiety. The prevalence of math anxiety is high across countries and age groups, and its consequences of math anxiety are far-reaching (Ma, 1999; Lee, 2009; Vukovic et al., 2013). People who suffer from higher math anxiety tend to avoid math, math-related environments and careers, particularly those involving science, technology, engineering and math throughout their lives (Meece et al., 1990; Chipman et al., 1992). Therefore, math anxiety can have a significant impact on people’s future career choices (Levy et al., 2021).

### Math self-efficacy

Improving children’s math self-efficacy and increasing their confidence in learning mathematics are critical to their future mathematical development (Kaskens et al., 2020; Casinillo, 2023; Kyaruzi, 2023). Mathematical self-efficacy is defined as an individual’s belief in their ability to perform mathematical tasks and is considered a predictor of math anxiety and performance (Hackett, 1985; Pajares and Miller, 1994; Bandura, 2012; Son et al., 2017). Gender differences have been found in mathematical self-efficacy (Kyaruzi, 2023), with men typically reporting higher levels than women (Hackett and Betz, 1981; Frenzel et al., 2010). Additionally, culture differences have been noted, with students from Asian countries such as Korea and Japan exhibiting low math self-efficacy and high levels of math anxiety despite high performance in mathematics (Lee, 2009).

### Parenting styles

Parents are key socializers and role models for their children, and their beliefs and parenting styles can influence their children’s learning and achievement in math (Maloney et al., 2015; Chang and Beilock, 2016). Parental academic stress and support have been found to be negatively correlated with students’ math performance (Puklek Levpušček and Zupančič, 2009; Chiu, 2017). Moreover, parenting style is related to math anxiety. Parenting styles have been classified into three categories: authoritative, authoritarian, and permissive (Baumrind, 1966, 1971, 1978). Authoritarian parenting styles have been found to have a direct negative impact on math anxiety. Children with parents who used authoritarian parenting styles obtain lower math scores (Feldman and Wentzel, 1990; Chao, 1994; Weiss and Schwarz, 1996; Chiu, 2017). On the other hand, authoritative parenting styles can have a direct positive effect on math anxiety while also having an indirect negative effect through math self-efficacy (Macmull and Ashkenazi, 2019). These findings suggest that parenting styles may affect children’s math performance and math anxiety. The impact of parenting style on math anxiety might be strongly influenced by math self-efficacy.

However, due to cultural differences between Eastern and Western cultures, the classification of parenting styles cannot be directly applied to Chinese parenting. Therefore, an appropriate classification of parenting styles should be selected to investigate the relationship between parenting style, math anxiety, and math self-efficacy in China. Given emotions are a crucial factor in math anxiety research (Deci et al., 1994) and that the participants in this study are Chinese elementary school students, the current study aims to explore the relationship between different expressions of emotions in parenting and math anxiety. Additionally, Chinese students grow up in an environment that places a greater emphasis on family and blood ties. Thus, a final classification of parenting styles based on emotions was utilized. The impact of Chinese parents’ emotions in parenting on students’ anxiety in learning mathematics is one of the main concerns of this study. The basic psychological needs of individuals, including emotions, are significant research components in the field of math anxiety (Parsons et al., 1982; Chiu, 2017). By examining the effects of parenting styles and emotions on students’ math anxiety, this study can enrich the research field of math anxiety related to the basic psychological needs of individuals. Specifically, this study is the first to use the emotional dimensions of parenting styles to investigate the relationships and effects between parenting styles, math self-efficacy, and math anxiety among Chinese elementary school students.

In this study, parenting styles are divided into three emotional dimensions: rejection, emotional warmth, and over protection (Arrindell et al., 1999; Arrindell and Engebretsen, 2000; Arrindell et al., 2005). Rejection can take many different forms, such as verbal abuse, punishment, choosing other family members over the child, constant criticism and rejection (Arrindell et al., 1999), which is similar to need frustration (Moè et al., 2020) and authoritarian parenting styles (Baumrind, 2012). Emotional warmth, on the other hand, includes appropriate care, warmth, affection, inspiration, and praise for the child, which are examples of acts that foster a positive emotional climate (Arrindell et al., 1999). This is similar to need satisfaction (Moè et al., 2020) and authoritative parenting styles (Yaffe, 2020). Finally, over protection is characterized by exaggerated concern and worry, stress for the child’s safety, intrusive hostility, and excessive involvement with the child (Arrindell et al., 1999). The relationships between these three dimensions of parenting style and math self-efficacy and math anxiety were examined separately to better understand the affective influence of parenting style on math anxiety and math self-efficacy.

### The relationship between math self-efficacy, parenting style, and math anxiety

Parents play a crucial role in the development of their children’s math anxiety (Maloney et al., 2015; Chang and Beilock, 2016). Math anxiety is influenced by social factors such as parental involvement in education and parenting styles. Different parenting styles have varying effects on math anxiety, with both direct and indirect correlations. Individuals subjected to authoritarian parenting exhibited higher levels of math anxiety (Sepehrianazar and Babaee, 2014; Macmull and Ashkenazi, 2019). Conversely, the authoritative parenting style has significant direct positive and indirect negative effects on math anxiety. Specifically, authoritative parenting has a direct positive effect on math anxiety, but it is also positively related to math self-efficacy, which reduces math anxiety through the role of math self-efficacy (Macmull and Ashkenazi, 2019). Permissive parenting style is positively correlated with math anxiety, albeit to a lesser extent (Macmull and Ashkenazi, 2019). Based on prior research, hypothesis 1a posited that math anxiety would be significantly and positively associated with rejection and over protection, whereas it would be negatively associated with emotional warmth and math self-efficacy. Hypothesis 1b posited that emotional warmth would be positively related to math self-efficacy, while rejection and over protection would be negatively related to math self-efficacy.

Studies had shown that parents influence their children’s math self-efficacy when pertains to their abilities and confidence in handling math tasks (Armstrong, 1981; Parsons et al., 1982). Moreover, parents’ perceptions and expectations of their children’s mathematical success also affect their children’s mathematical abilities (Yee and Eccles, 1988). Students are expected to perform better academically when parents encouraged them to develop communication skills and independence while providing the necessary demands and boundaries for learning, which is a hallmark of authoritative parenting (Vansteenkiste et al., 2006). These students not only have higher grade point averages but also higher levels of academic confidence (Cetin, 2015). Clearly, parents, as primary influencers of their children’s learning, play a significant role in their math self-efficacy when learning mathematics. Examining the effects of parenting styles on children’s math self-efficacy would provide further insight into math self-efficacy and lay the groundwork for future improvements in children’s self-efficacy. Therefore, based on the above studies and considering the role of math self-efficacy between parenting style and math anxiety as described above, this study used math self-efficacy as a mediating variable to examine the relationship between parenting style and math anxiety. Together with the above-mentioned arguments, hypothesis 1c proposed that there would be a significant mediating effect of math self-efficacy between parenting style and math anxiety.

Anxiety among students in academic settings can lead to low self-efficacy (Usher and Pajares, 2008), as students perceive their anxiety as evidence of their lack of success (Bandura, 1977, 2012). Anxiety about mathematics can manifest as early as mid-primary school (Gierl and Bisanz, 1995). Studies with French elementary school students have found no link between math anxiety and self-efficacy (Joët et al., 2011). In contrast, studies of middle and high school students in the United States have found that those with greater math anxiety believed they were less effective at solving mathematical problems (Lopez and Lent, 1992; Lent et al., 1996; Lopez et al., 1997). According to Galla and Wood (2012), math self-efficacy played a positive role in reducing the negative effects of anxiety.

### Aims and hypotheses

In this study we collected data from 454 fifth and sixth-grade students to investigate the associations among math self-efficacy, parenting style, and math anxiety in primary school children in China. Drawing upon the complex relationship between parenting styles, math self-efficacy, and math anxiety described above, this study develops the following hypotheses based on prior research: (H1a) Emotional warmth and math self-efficacy are significantly negatively associated with math anxiety, whereas rejection and over protection were significantly positively associated with math anxiety; (H1b) Emotional warmth is significantly positively related to math self-efficacy, while rejection and over protection were significantly negatively related to math self-efficacy; (H1c) There would be a significant mediating effect of math self-efficacy between parenting style and math anxiety. Meanwhile, since previous research has identified gender differences in math self-efficacy (Macmull and Ashkenazi, 2019) and math anxiety (Hopko et al., 2003; Beilock et al., 2010; Bieg et al., 2015; Dowker et al., 2016) in previous studies, and this study also aims to further investigate gender differences in these constructs by proposing Hypothesis 2 that math self-efficacy and math anxiety will significantly differ by gender.

## Materials and methods

### Participants

A total of 454 fifth- and sixth-grade students from a public elementary school in Zhejiang Province, China, were selected to participant in this study. Participants with a significant amount of missing data were excluded from the analysis. Missing data was considered significant when there were unanswered questions in any questionnaire that accounted for more than 20% of the total number of questions. Furthermore, data points that exceeded 1.98 standard deviations from the mean were also excluded from the analysis. Ultimately, 400 valid participants’ data were obtained, resulting in effective response rate of 88.11%. Of the participants, 197 were boys and 203 were girls, with 201 in the fifth grade and 199 in the sixth grade. All students participated in this study only after receiving informed consent from their legal guardians and providing their own oral consent.

### Procedure

The research team first submitted the scales used in the study to the school director for review. The director evaluated whether the meaning of the question items in the scale was acceptable to students and whether the wording of the items was positive and avoided any adverse psychological effects on the students. After the director approved the scales and determined the time for distribution, the scales were administered and collected in the classroom. The three scales were answered by students. The main test was administered by experienced graduate students in psychology and education, and a guideline was read out by the main examiner during the test. The collected data was analyzed using SPSS 21.0.

### Measures

#### Math anxiety rating scale

The mathematics anxiety scale used in this study was based on the Mathematics Anxiety Rating Scale developed by Richardson and Suinn (1972). It was developed specifically for the mathematics discipline and elementary school students. The scale consisted of 27 self-report items, with items 24, 25, and 26 items reversed scored. The scale measured four dimensions of math anxiety: stress fear, emotional worry, test anxiety, and classroom anxiety, using a five-point Richter scale. A score of 1 represented very non-conforming, 2 represented non-conforming, 3 represented partially conforming, 4 representing conforming, and 5 represented very conforming. A higher rating on the scale corresponded to higher math anxiety. The total score for each dimension was divided by the total number of items for that dimension, and the resulting mean score was the score for that dimension. Higher scores indicated greater anxiety for each dimension and overall math anxiety. The internal consistency reliability Cronbach’s α for the stress fear, emotional worry, test anxiety, and classroom anxiety dimensions were 0.85, 0.84, 0.81, and 0.78, respectively, while the internal consistency reliability Cronbach’s α for the overall scale was 0.93.

#### The revised short-form parenting style scale

The Revised Short-Form Parenting scale was based on Arrindell’s scale (Arrindell et al., 1999, 2005; Arrindell and Engebretsen, 2000), with modifications made to localize it to the Chinese context. The scale was known as the Short-Enga Minnen Barndoms Uppfostran Chinese Version (s-EMBU-C) and was a self-administered questionnaire divided into a father’s version and a mother’s version, each with 21 items and identical content. The 15th item was reverse scored, and the scale measured three dimensions: rejection, emotional warmth, and over protection. A four-point Richter scale was used, with a score of 1 indicating “never,” 2 indicating “occasionally,” 3 indicating “often,” and 4 indicating “always.” The total score of each dimension was divided by the total number of questions in that dimension, and the average score was the final score of that dimension. A higher score on each dimension indicated a greater likelihood that the student had experienced that specific parenting style, or that the child felt the degree of the dimension in their relationship with their parents. The internal consistency coefficient of the revised short-form parenting style warmth ranged from 0.74 to 0.84, and the retest reliability after 10 weeks ranged from 0.70 to 0.81, indicating good reliability (Jiang et al., 2010).

#### Math self-efficacy scale

The math self-efficacy scale used in this study was adapted from the relevant dimensions of the Teacher Efficacy Scale, originally developed by Gibson and Dembo (1984). The self-report scale consisted of 12 items, with items 5, 6, and 9 being reversed scored. The scale included two dimensions: the first six items measured Mathematical Ability Self-Efficacy, while the last 6 items were Mathematical Behavior Self-Efficacy. Participants rated their level of agreement with each item on a five-point Richter scale (1 = totally disagree; 3 = generally; 5 = totally agree). To calculate scores of each dimension, the total score for each dimension was divided by the number of items in that dimension, and the resulting average score was taken as the score for that dimension. The internal consistency coefficient of the scale was 0.85 (Chen and Wang, 2018). In this study, the internal consistency reliability Cronbach’s α for the Mathematical Ability Self-efficacy dimension and the Mathematical Behavior self-efficacy dimension were 0.82 and 0.85, respectively.

### Statistical analyses

To investigate the relationship between parenting style, math self-efficacy and math anxiety among fifth and sixth-grade students, descriptive statistics and Pearson correlation analysis was performed. Furthermore, in order to further examine the mediating role of math self-efficacy, this study utilized rejection and emotional warmth as predictor variables, with math anxiety as the outcome variable and math self-efficacy as the mediating variable. The SPSS PROCESS component was employed and Model 4 was selected for the analysis of mediating effects, which was a full mediation model. To examine the mediating effects, the bias-corrected nonparametric percentile Bootstrap method created by Hayes (2013) was utilized. Specifically, 95% confidence intervals were calculated for each of the 5,000 replicate samples, and statistical significance was indicated if the confidence interval did not contain a value of 0.

## Results

### Descriptive statistics for each variable

Table 1 showed the descriptive statistics. The results of the correlation analysis (Table 1) indicated that emotional warmth and math self-efficacy had a significant negative correlation with math anxiety, while rejection had a significant positive correlation with math anxiety. Emotional warmth also had a significant positive correlation with math self-efficacy, while rejection had a significant negative correlation with math self-efficacy. In addition, over protection had a significant negative correlation with emotional warmth, and a significant positive correlation with rejection. Finally, there was a significant negative correlation between rejection and emotional warmth.

**Table 1:** Means, standard deviations, and correlations between all the variables.

	Mean	SD	Rejection	Emotional warmth	Over protection	Math self-efficacy	Math anxiety
Rejection	1.32	0.36	1				
Emotional warmth	3.15	0.58	−0.52^**^	1			
Over protection	1.97	0.44	0.41^**^	−0.22^**^	1		
Math self-efficacy	3.72	0.79	−0.12^*^	0.33^**^	−0.01	1	
Math Anxiety	2.17	0.79	0.19^**^	−0.27^**^	0.07	−0.75^**^	1

^*^p < 0.05, ^**^p < 0.01, ^***^p < 0.001 for bold values.

The results suggested that there was a strong association between parenting style, math self-efficacy, and math anxiety among fifth and sixth-grade students. The findings partly supported Hypothesis 1a, which proposed a significant negative relationship between emotional warmth, math self-efficacy and math anxiety, as well as a significant positive relationship between rejection and math anxiety. However, there was no significant correlation between over protection and math anxiety. The results also partially support Hypothesis 1b, which posited a significant positive correlation between emotional warmth and math self-efficacy, a significant negative correlation between rejection and math self-efficacy, and no significant correlation between over protection and math self-efficacy.

Gender differences were considered in this study, and the correlation between parenting style, math anxiety, and math self-efficacy for male and female students were analyzed separately (Table 2). Results showed that there were significant gender differences in the correlations between rejection and math self-efficacy, as well as between over protection and math anxiety. Specifically, the correlation between rejection and math self-efficacy was insignificant for male students, whereas it was significant for female students. Similarly, the correlation between over protection and math anxiety was insignificant for male students, while it was significant for female students.

**Table 2:** Means, SDs, and correlations between all the variables separated by gender.

	M_G_ (SD_G_)	M_B_ (SD_B_)	Rejection	Emotional warmth	Over protection	Math self-efficacy	Math anxiety
Rejection	1.33 (0.37)	1.32 (0.35)	1	−0.55^**^	0.46^**^	−0.15^*^	0.22^**^
Emotional warmth	3.08 (0.60)	3.22 (0.55)	−0.49^**^	1	−0.28^**^	0.32^**^	−0.25^**^
Over protection	1.88 (0.43)	2.06 (0.42)	0.38^**^	−0.24^**^	1	−0.13	0.18^*^
Math self-efficacy	3.55 (0.77)	3.89 (0.78)	−0.08	0.32^**^	−0.02	1	−0.75^**^
Math anxiety	2.36 (0.82)	1.97 (0.70)	0.16^*^	−0.25^**^	0.07	−0.73^**^	1

MG refers to the mean value of boys and MB refers to the mean value of girls, while SDG and SDB refer to the standard deviation values of girls and boys, respectively. ^*^p < 0.05, ^**^p < 0.01, ^***^p < 0.001 for bold values. The data for female students is above the table’s diagonal line, while the data for male students is below it.

In addition, the results of the independent samples t-test showed significant gender differences in emotional warmth [*t*(399) = 2.52, *p* < 0.05], over protection [*t*(399) = 4.20, *p* < 0.01], math self-efficacy [*t*(399) = 4.48, *p* < 0.01], and math anxiety [*t*(399) = −5.08, *p* < 0.01] among fifth and sixth-grade elementary school children. These results support Hypothesis 2. Boys showed higher emotional warmth compared to girls, while boys were higher in over protection compared to girls (Table 2). Moreover, boys reported higher math self-efficacy compared to girls, and boys exhibited lower math anxiety than girls (Table 2). No significant gender difference was found in rejection [*t*(399) = −0.07, *p* > 0.05].

### An analysis of the mediating role of math self-efficacy on the relationship between parenting style and math anxiety

**Figure 1:**
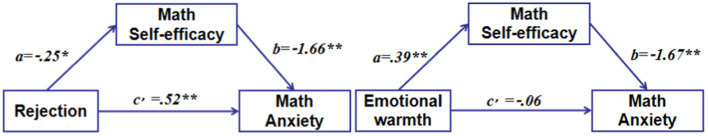
Analysis of the mediating role of math self-efficacy on the relationship between rejection and math anxiety (left) and the Relationship between emotional warmth and math anxiety (Right). ^*^p < 0.05; ^**^p < 0.01.

The results of this analysis were summarized in Table 3. The results showed that the indirect effect of rejection on math anxiety through math self-efficacy was significant, as the 95% confidence interval did not include zero. Moreover, the mediating effect was found to be 44.68% of the total effect. The indirect effect of emotional warmth on predicting math anxiety through math self-efficacy was significant, as the 95% confidence interval did not include zero, with the proportion of the mediating effect to the total effect being 91.55%. However, the indirect effect of over protection on predicting math anxiety through math self-efficacy was not significant, as the 95% confidence interval included zero. Therefore, Hypothesis 1c was partly supported. Furthermore, the results of this study indicated that there was a significant mediating effect of math self-efficacy between rejection and emotional warmth and math anxiety. However, there was no significant mediating effect of math self-efficacy on the relationship between over protection and math anxiety (Figure 1).

**Table 3:** Analysis of the mediated effects between parenting style and math anxiety (*N* = 400).

Parenting style	Mediating variable	Total effect value	Indirect effect value	Boot SE	Boot CI lower limits	Boot CI upper limits	Intermediation effect values (%)
Rejection	Math self-efficacy	0.94	0.42	0.19	0.05	0.79	44.68
Emotional warmth	Math self-efficacy	−0.71	−0.65	0.10	−0.85	−0.46	91.55
Over protection	Math self-efficacy	0.22	0.02	0.12	−0.20	0.26	-

**Figure 2:**
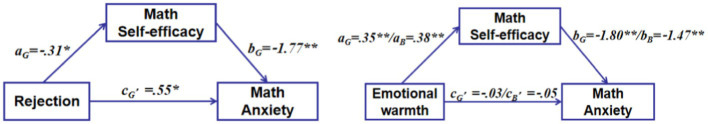
Mediating role of math self-Efficacy on relationship between rejection and math anxiety for girls only (left), and emotional warmth and math anxiety for boys and girls (right). a_G_, b_G_, and c’_G_ represent the coefficients for girls. a_B_, b_B_, and c’_B_ represent the coefficients for boys.^*^p < 0.05; ^**^p < 0.01. In the above figure, coefficients a and b were significant while c’ was not, indicating that math self-efficacy had a fully mediating role in emotional warmth and math anxiety.

We further analyzed the data separately for male and female students to examine the gender differences, and found that there were gender differences in the mediating effects. The results for female students were consistent with the overall analysis (Table 4), while for male students, math self-efficacy only mediated the relationship between emotional warmth and math anxiety (Table 5; Figure 2).

**Table 4:** Analysis of the mediated effects between parenting style and math anxiety for girls (*N* = 203).

Parenting style	Mediating variable	Total effect value	Indirect effect value	Boot SE	Boot CI lower limits	Boot CI upper limits	Intermediation effect values (%)
Rejection	Math self-efficacy	1.09	0.54	0.26	0.01	1.05	49.54
Emotional warmth	Math self-efficacy	−0.65	−0.62	0.14	−0.90	−0.35	95.38
Over protection	Math self-efficacy	0.57	0.30	0.18	−0.02	0.66	-

**Table 5:** Analysis of the mediated effects between parenting style and math anxiety for boys (*N* = 197).

Parenting style	Mediating variable	Total effect value	Indirect effect value	Boot SE	Boot CI lower limits	Boot CI upper limits	Intermediation effect values (%)
Rejection	Math self-efficacy	0.74	0.27	0.24	−0.19	0.78	-
Emotional warmth	Math self-efficacy	−0.62	−0.56	0.13	−0.84	−0.32	90.32
Over protection	Math self-efficacy	0.21	−0.05	0.14	−0.32	0.25	-

## Discussion

This study examined the relationship among parenting styles, math self-efficacy, and math anxiety in primary school students. The study revealed significant relationships between parenting styles, math self-efficacy, and math anxiety. Specifically, students who experienced more rejection from their parents reported higher level of math anxiety. Moreover, emotional warmth in parenting was found to be significantly negatively correlated with math anxiety, meaning that the higher level of emotional warmth perceived by children, the lower level of math anxiety they would perceive. Additionally, there was a negative correlation between math self-efficacy and math anxiety, suggesting that an increase in math self-efficacy led to a decrease in math anxiety. The study also found gender differences in math anxiety and math self-efficacy, with females displaying higher levels of math anxiety and lower levels of math self-efficacy compared to males.

Finally, the study showed that math self-efficacy had a significant mediating effect on the relationship between rejection and math anxiety, as well as on the relationship between emotional warmth and math anxiety. However, there was no significant mediating effect of math self-efficacy on the relationship between over protection and math anxiety.

### The relationship between math self-efficacy, parenting style, and math anxiety

Griggs et al. (2013) suggested parents played a significant role in influencing their children’s self-efficacy, as parents were the primary individuals with whom children interact. When parents adopted a rejecting parenting style, children may struggle to acquire problem-solving skills, adjust to daily life, evaluate situations, and develop appropriate action plans with parental guidance and experience. Over time, this may lead to deficiency in problem-solving abilities, which could resulted in heightened anxiety and reduced self-efficacy (Rapee, 1997; Chorpita and Barlow, 1998; Wood et al., 2003).

When children experienced rejection, they may exhibit cautious behavior in school and became overly attentive to cues presented in the school environment (Sherman et al., 2013). This may be particularly pronounced in situations where there were many students, and teachers were unable to provide individualized attention, as such behavior may be perceived by the child as rejection. Future research could explore the potential relationship between parental rejection and students’ perceptions of teacher rejection, as well as how both factors may influence math anxiety and math self-efficacy among students.

In order to foster the development of children’s self-efficacy, emotional warmth from parents is essential (Zimet et al., 1988). Emotional warmth refers to the ability of parents to create a warm and supportive environment for their children, in which they can act autonomously, make their own choices, and express their own perspectives (Turner et al., 2009). When parents adopt this approach, it can have a positive impact on children’s self-efficacy, which in turn can help them to feel more confident in using their own strategies to learn math. This may not always lead to immediate success, but over time, it can help to build children’s self-efficacy and reduce their anxiety around math. By creating a nurturing environment that fosters children’s self-efficacy, parents can help their children to become more engaged and effective learners in math.

In prior research, the concept of over protection was similar to the authoritarian parenting style, wherein parents exhibit excessive interference in their children’s lives as per their preferences (Macmull and Ashkenazi, 2019). However, unlike earlier studies, no correlation was observed between over protection and math anxiety in this study. Earlier studies have shown that an authoritarian parenting style leads to an increase in children’s anxiety, and the mediation effect of such a parenting style was significant between math self-efficacy and math anxiety (Macmull and Ashkenazi, 2019). The authoritarian parenting style is known to render children passive learners with low self-efficacy, consequently hampering their academic abilities (Diener and Dweck, 1978; Macmull and Ashkenazi, 2019). Nonetheless, the present study conducted in China did not observe a significant correlation between over protection and math anxiety. The findings could be attributed to the influence of the Chinese culture, where children are accustomed to excessive help and protection from their parents, and grow up in a structured and parent-dominated environment.

### Gender differences in math anxiety and math self-efficacy

Gender differences in math anxiety and math self-efficacy have been widely reported in the literature (Else-Quest et al., 2010; Hill et al., 2016; Su et al., 2021). Prior studies have consistently found that females reported higher levels of math anxiety and lower levels of math self-efficacy compared to males. The current findings are consistent with these prior reports. These findings underscore the importance of addressing gender differences in math anxiety and self-efficacy, as well as the need to support students in managing stress and anxiety in the classroom, particularly as they progress through their academic careers.

The gender differences were found not only in the levels of math anxiety and math efficacy, but also in the significance of the correlations between rejection and self-efficacy and over protection and math anxiety. These results suggest that cultural factors may play a role in shaping these gender differences. Specifically, in the context of Chinese family education and social culture, boys may experience more rejection from parents due to the perception that they are more active and trouble-prone than girls. This may explain why the correlation between rejection and math self-efficacy was not significant for boys. In contrast, girls may experience less rejection and more praise for being well-behaved, which could explain why they may be more affected by rejection behaviors that impact their math self-efficacy. Gender stereotypes related to personality and ability are not unique to China and have been studied in other cultural contexts (Tomasetto et al., 2011; Ceci, 2018; Moè et al., 2021). The relationship between rejection and math self-efficacy may not be solely tied to gender, but also to the personality traits. Therefore, exploring the connections between rejection, gender, and math self-efficacy in various cultural contexts could provide additional insights into these relationships.

Over protection was significantly and positively associated with girls’ math anxiety, while it was not significantly associated with boys’ math anxiety. This may due to the fact that in China, boys tend to exhibit contradictory behaviors. On one hand, they may be troublemakers and disruptive in the family, while on the other hand, they may enjoy having their parents clean up after them. As a result, boys may view over protection as normal behavior, and may not experience a significant correlation between over protection and math anxiety. On the other hand, girls are often encouraged to be well-behaved, which may lead to them relying more on their own judgment and ideas to conduct their lives. Parental over protection may then make them feel controlled and dominated, resulting in a significant positive correlation with girls’ math anxiety.

The study also found gender differences in the analysis of mediating effects. Math self-efficacy was found to be significantly mediated only between emotional warmth and math anxiety in male students. The lack of significant mediation between rejection and math anxiety can be attributed to the fact that Chinese boys, as discussed earlier, experience more rejection behaviors while growing up compared to Chinese girls, which makes them more accustomed to rejection and less affected by it in terms of their math self-efficacy.

This study not only found gender differences in the perceptions of emotional warmth, over protectiveness, math self-efficacy, and math anxiety among boys and girls but also revealed differences in how boys and girls perceive the relationships among these variables. Gender differences often mask socio-cultural gender stereotypes, such as the stereotype that boys are more active and girls are more obedient. Both males and females face gender stereotypes and socio-cultural constraints that restrict them to conform to narrow socio-cultural biases. In STEM education, for instance, research has found that there exists a stereotype that females perform poorly compared to males (Moè et al., 2021). Alleviating the anxiety that exists in the area of mathematics learning is an important part of STEM education, which can help learners overcome gender stereotypes prevalent in STEM learning.

### The mediating effect of mathematical self-efficacy in the relationship between parental rejection and emotional warmth and mathematical anxiety

The present study demonstrated a significant mediating effect of mathematical self-efficacy in the relationship between parental rejection and emotional warmth and mathematical anxiety. Specifically, the results suggested that the degree of perceived rejection by the child during parent–child interactions was negatively associated with mathematical self-efficacy, which in turn was positively associated with higher levels of mathematical anxiety. Conversely, higher levels of perceived emotional warmth were positively associated with mathematical self-efficacy, which in turn was negatively associated with higher levels of mathematical anxiety. These findings may be explained by the fact that children who perceive higher levels of rejection during parent–child interactions may develop lower levels of mathematical self-efficacy, which may lead to increased levels of mathematical anxiety. On the other hand, children who perceive higher levels of emotional warmth may develop higher levels of mathematical self-efficacy, which may lead to decreased levels of mathematical anxiety. Previous research has also suggested that students who perceive that their parents provide them with opportunities to develop their communication skills, autonomy, and set clear boundaries tend to perform better academically and exhibit higher levels of self-efficacy (Turner et al., 2009). These findings underscore the importance of promoting positive parent–child interactions, particularly with regard to fostering emotional warmth and reducing perceptions of rejection, as a means of enhancing mathematical self-efficacy and reducing mathematical anxiety in children. Such efforts may be particularly important for children who are at risk of developing low levels of mathematical self-efficacy and high levels of mathematical anxiety.

## Conclusion and practical implications

The results of this study indicate that emotional warmth is positively associated with children’s math self-efficacy, highlighting the importance of creating a warm and supportive family environment to reduce children’s math anxiety. Parents should provide their children with encouragement, support, and assistance to help them develop their math self-efficacy and minimize rejection, which can damage their sense of self-efficacy. Teachers can also play a crucial role in creating a warm and supportive learning environment that fosters students’ self-efficacy and reduces math anxiety. To alleviate math anxiety in children, it is essential to create an environment that promotes their sense of self-efficacy. Parents and teachers can create this environment by offering praise and encouragement, providing opportunities for success, and reinforcing their children’s self-beliefs. Moreover, parents can help their children develop communication skills and autonomy, while setting appropriate boundaries to work within. Teachers can use instructional strategies that build students’ self-efficacy, such as scaffolding, modeling, and providing opportunities for practice and feedback. Overall, the findings of this study suggest that creating a warm and supportive environment at home and school can help to alleviate math anxiety in children. By promoting children’s math self-efficacy, parents and teachers can help students feel more confident and competent in math-related subjects, which may ultimately lead to improved academic performance and a greater enjoyment of mathematics.

Given the gender differences between boys and girls, it is crucial to adopt a more open and inclusive mindset that respects these differences in education and upbringing. Boys and girls should not be limited to societal and cultural constructs, but allowed to express their authentic selves. For instance, girls can be lively and boys can be well-behaved, which can help them reach their full potential.

## Limitations

The study population comprised Chinese fifth and sixth graders, and future research should aim to expand upon the investigation of the mediating relationship between parenting style, math self-efficacy, and math anxiety. Additionally, as the sample size grows larger, it would be worthwhile to examine whether there are any grade-based differences in the mediating relationship as identified in the present study. This study utilized student self-reports as the sole data collection method. Future research should consider utilizing multiple data sources, such as parental reports on parenting styles. When assessing students’ math self-efficacy or anxiety, feedback evaluations from peers and math teachers should also be considered. Additionally, to gain a better understanding of the relationship between parenting styles, math self-efficacy, and math anxiety, future research could employ a longitudinal design to examine how each variable changes and interacts over time.

## Data availability statement

The raw data supporting the conclusions of this article will be made available by the authors, without undue reservation.

## Ethics statement

The studies involving human participants were reviewed and approved by Huzhou University. The patients/participants provided their written informed consent to participate in this study.

## Author contributions

CW and XL: conceptualization. CW: methodology, supervision, and funding acquisition. H-jW, CW, and XL: validation. XL: formal analysis, writing—original draft preparation, and visualization. CW and H-jW: writing—review and editing. H-jW: project administration. All authors contributed to the article and approved the submitted version.

## Funding

This research was funded by Humanity and Social Science Youth foundation of Ministry of Education of China, grant number 22YJCZH164 and Graduate Research and Innovation Funding from the School of Teacher Education, Huzhou University (2022JSJYYK07).

## Conflict of interest

The authors declare that the research was conducted in the absence of any commercial or financial relationships that could be construed as a potential conflict of interest.

## Publisher’s note

All claims expressed in this article are solely those of the authors and do not necessarily represent those of their affiliated organizations, or those of the publisher, the editors and the reviewers. Any product that may be evaluated in this article, or claim that may be made by its manufacturer, is not guaranteed or endorsed by the publisher.
